# Editorial: Methods on the Assessment of Human Baroreflex Function

**DOI:** 10.3389/fnins.2022.965406

**Published:** 2022-06-28

**Authors:** Yue-Der Lin, André Diedrich

**Affiliations:** ^1^Department of Autonomic Control Engineering, Feng Chia University, Taichung, Taiwan; ^2^Master's Program of Biomedical Informatics and Biomedical Engineering, Feng Chia University, Taichung, Taiwan; ^3^Vanderbilt Autonomic Dysfunction Center, Vanderbilt University Medical Center, Nashville, TN, United States; ^4^Department of Biomedical Engineering, Vanderbilt University, Nashville, TN, United States

**Keywords:** blood pressure, heart rate, autonomic nervous system, baroreflex, sympathetic activity, vagal activity

The human baroreflex represents a crucial integrative neuronal negative feedback system which confines excessive blood pressure fluctuations into physiologically normal ranges (Robertson et al., 2012). Arterial baroreceptors within carotid sinuses, the aortic arch, and receptors in cardiopulmonary veins and right atrium sense pressure changes and transmit input through the glossopharyngeal nerve and vagus nerve to *nucleus tractus solitarii* (NTS) in the brainstem. During high blood pressure, baroreceptors respond to vascular wall stretch, which inhibits sympathetic pacemaker neuron in the rostral ventrolateral medulla (RVLM) via an inhibotry neuron in the caudal-ventrolateral medulla (CVLM). At the same time, excitatory projections from NTS increases cardiac parasympathetic drive from nucleus ambiguus (NA) and the dorsal motor nucleus of the vagus (DMNV), in turn increasing release of acetylcholine at the sinoatrial node and decreasing heart rate (details in Figure 1). During upright, blood pressure falls and unloads vascular wall, thereby reducing the activity of the baroreceptor afferent nerves which leads to decrease in cardiac vagal tone and increase in sympathetic activity. Sympathetic vasoconstrictor drive initiates release of norepinephrine from the sympathetic nerve terminals on the blood vessels. It causes both arterial vasoconstriction and venoconstriction and an increase in cardiac contractility and heart rate, all of which contribute to normalization of arterial pressure and cerebral perfusion (Figure 1).

**Figure 1:**
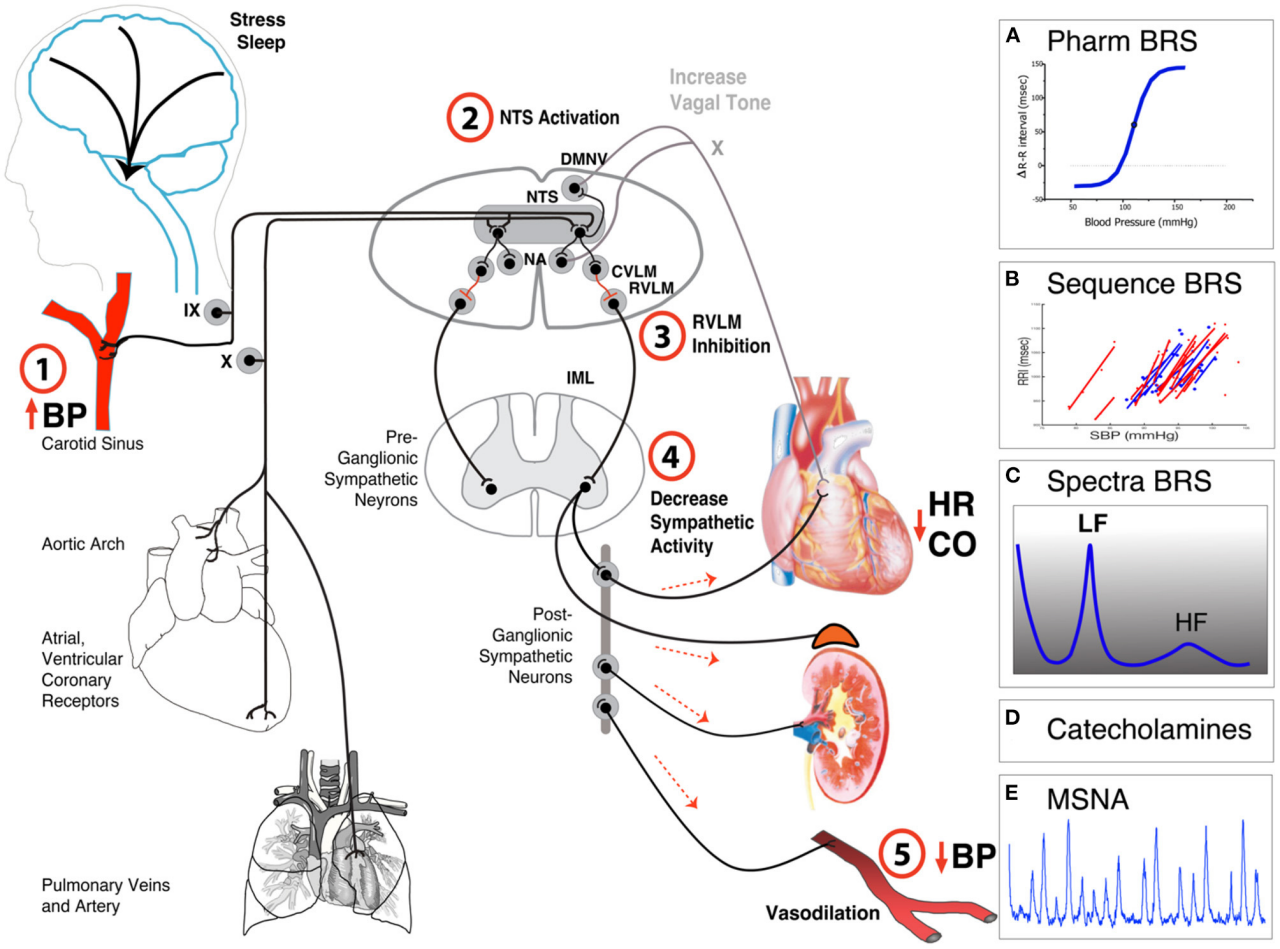
Simplified anatomical and functional scheme of baroreflex function. Baroreceptors in the carotid sinus, aortic arch, right atrium and in the cardiopulmonary veins are activated-by (1) stretching and passing this information through the Vagus (X) or glossopharyngeal (IX) nerves to (2) the nucleus tractus solitarii (NTS) of the brain stem. The NTS projects to neurons in caudal ventrolateral medulla (CVLM) which (3) inhibits sympathetic pacemaker neurons in the rostral ventrolateral medulla (RVLM) and (4) reduces sympathetic outflow. This decreases heart rate, cardiac output, and (5) blood pressure. Activation of the NTS projects also to the nucleus ambiguous (NA) and dorsal motor nucleus of the Vagus (DMNV) and increases parasympathetic activity. Common methods to evaluate baroreflex function include **(A)** pharmacological challenge with vasoactive drugs, **(B)** spontaneous sequence analysis, **(C)** spectral analysis, **(D)** measurements of catecholamines, or **(E)** muscle sympathetic activity (MSNA) during orthostatic challenges.

Baroreflex lesions can affect the afferent limb, the peripheral limb, or the central integration centers. The clinical picture of patients with afferent baroreflex failure is characterized by unpredictable hypertensive crises, symptomatic hypotensive episodes, and orthostatic hypotension, demonstrating the importance of the baroreflex (Biaggioni et al., 2019). Patients with pure autonomic failure with postganglionic lesions experience disabling orthostatic hypotension. The correct assessment of human baroreflex is important not only for targeted treatment of autonomic dysfunction but also for prediction of cardiovascular risk in heart failure, hypertension, diabetes, and other cardiovascular diseases.

This Research Topic focuses on baroreflex measurements in humans. Baroreflex methods can be divided in pharmacological and non-pharmacological testing. Non-pharmacological testing includes measurements of baroreflex responses during neck suction, Valsalva maneuver, controlled breathing, exercise, and orthostatic stress. In addition, baroreflex sensitivity (BRS) can be assessed using analysis of blood pressure and heart rate variability, neural sympathetic activity, and modeling (Figure 1). Traditional baroreflex methods assume that the baroreflex system is an open loop system, but that is not the case for living human subjects. In animal studies, the closed loop can be opened surgically and pharmacologically. In humans, measurements in closed loop system are difficult. Pharmacological testing using vasoactive substances underestimate the effects, as the overall effect is determined by a combination of the direct peripheral effect on vascular tone and the indirect central baroreflex buffering (Jordan et al., 2002).

In this Research Topic, new methods for closed loop identification of baroreflex properties have been discussed. Kawada et al. described a method using white noise perturbation to measure central and peripheral arc properties. Yamasaki et al. proposed a novel analytical framework using equilibrium diagram to overcome the close loop open loop problem.

Measurement of baroreflex function using stimuli is the preferred approach, and it has some advantages in comparison to analysis of spontaneous fluctuations caused by biological noise. Neck suction is a direct non-pharmacological way to stimulate carotid baroreceptor and measure the physiological response. This complex technique is not available in the clinic. Pinheiro et al. developed an improved noiseless neck suction chamber device for use in clinic. Huang et al. used the Valsalva maneuver, and they compared it with spontaneous sequences method and BRS estimation using spectral methods. Dutra-Marques et al. used exercise as a stimulation to measure baroreflex response.

This Research Topic also includes improvement of traditional methods of baroreflex slope estimation in addition to novel approaches, such as analysis of baroreflex hysteresis, cross-correlations, and analysis of synchronization between heart rate and blood pressure. Heusser et al. derived a modified curve fitting model of the sigmoidal baroreflex curve, directly obtaining the baroreflex curve slope and other meaningful physiological parameters. Dabiri et al. used a novel ellipse analysis to characterize hysteresis of the spontaneous respiration-related cardiovagal baroreflex for orthostatic test. The proposed method, by considering gain and set-point changes during respiration, offers instructive insight into the resulting hysteresis of the spontaneous cardiovagal baroreflex with respiration as stimuli.

The influence of higher nervous activity on the cardiovascular system via autonomic control and baroreflex regulation has been neglected for a long time. This is especially the case in sleep, as baroreflex properties change during different sleep stages. Karavaev et al. proposed a quantitative measure representing the total percentage of phase synchronization between the low-frequency oscillations in heart rate and blood pressure. They showed that the degree of synchronization of the studied rhythms is higher in slow-wave sleep compared to the awake state but lower compared to the rapid eye movement sleep.

This Research Topic also presents new methods in detecting cardiovascular autonomic neuropathy in patients with Parkinson's disease, diabetes, and metabolic syndrome. Huang et al. compared spontaneous sequence and spectral BRS with baroreflex slope determined using the Valsalva maneuver in Parkinson's Disease. Yeh et al. proposed an advanced cross-correlation function method with adjustable threshold which was superior for early detection of autonomic neuropathy in diabetes mellitus. Dutra-Marques et al. compared spontaneous sequential baroreflex measures with the exercise responses in patient with metabolic syndrome to study baroreflex mechanisms and risks factors of metabolic syndrome.

Our hope is that readers will gain new insight of novel concepts and measures of baroreflex function. We thank all authors for their contribution to this area of research, as it demonstrates the importance of developing novel methods of baroreflex measures for the early detection of dysfunction and the improvement of treatment of our patients.

## Author Contributions

The draft was written by AD and proofread and approved by Y-DL. Both authors contributed to the article and approved the submitted version.

## Funding

This research was funded by Ministry of Science and Technology, Taiwan (contract number: MOST 110-2221-E-035-006-MY3). AD was partly supported by NIH R01 HL142583.

## Conflict of Interest

The authors declare that the research was conducted in the absence of any commercial or financial relationships that could be construed as a potential conflict of interest.

## Publisher's Note

All claims expressed in this article are solely those of the authors and do not necessarily represent those of their affiliated organizations, or those of the publisher, the editors and the reviewers. Any product that may be evaluated in this article, or claim that may be made by its manufacturer, is not guaranteed or endorsed by the publisher.
